# Supplementary material for: The interactome of intact mitochondria by cross-linking mass spectrometry provides evidence for coexisting respiratory supercomplexes
Source: Mol Cell Proteomics. 2017 Dec 8;17(2):216–32. doi: 10.1074/mcp.RA117.000470 (PMC5795388; doi:10.1074/mcp.RA117.000470)
Supplement: Supplemental Data [file supp_RA117.000470_133922_0_supp_23978_fzffwf.zip › spectra_annotation/mito_spectra_annotation/348-1-10-1-4-1.pdf]

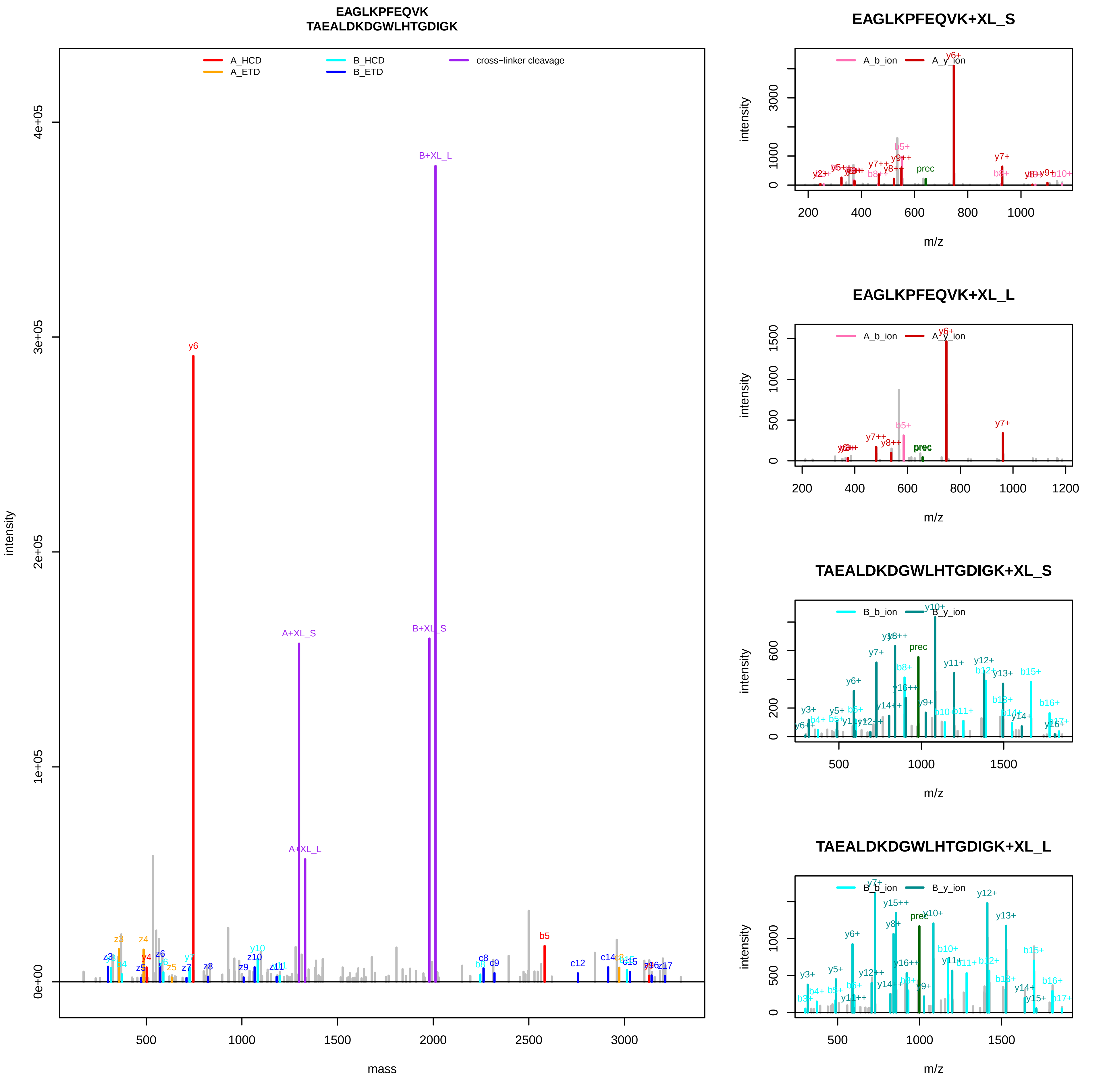
EAGLKPFEQVK
TAEALDKDGWLHTGDIGK
A_HCD
A_ETD
B_HCD
B_ETD
cross−linker cleavage
B+XL_L
y6
A+XL_S
B+XL_S
A+XL_L
b5
intensity
mass
0e+00
1e+05
2e+05
3e+05
4e+05
500
1000
1500
2000
2500
3000
EAGLKPFEQVK+XL_S
A_b_ion
A_y_ion
y6+
y7+
b5+
prec
intensity
m/z
EAGLKPFEQVK+XL_L
A_b_ion
A_y_ion
y6+
y7+
b5+
prec
intensity
m/z
TAEALDKDGWLHTGDIGK+XL_S
B_b_ion
B_y_ion
y10+
y7+
y6+
prec
b8+
y11+
y12+
y13+
b15+
b16+
intensity
m/z
TAEALDKDGWLHTGDIGK+XL_L
B_b_ion
B_y_ion
y7+
y12+
y15++
y10+
y13+
y8+
prec
y6+
b15+
intensity
m/z
